# Supplementary material for: Advancing crop classification in smallholder agriculture: A multifaceted approach combining frequency-domain image co-registration, transformer-based parcel segmentation, and Bi-LSTM for crop classification
Source: PLoS One. 2024 Mar 1;19(3):e0299350. doi: 10.1371/journal.pone.0299350 (PMC10906854; doi:10.1371/journal.pone.0299350)
Supplement: Attachment — Submitted filename: Response to Reviewers.docx [file pone.0299350.s001.docx]

Original Manuscript ID: **PONE-D-23-40002**

Original Article Title: “**Advancing Crop Classification in Smallholder Agriculture: A Multifaceted Approach Combining Frequency-Domain Image Co-Registration, Transformer-Based Parcel Segmentation, and Bi-LSTM for Crop Classification**”

To: PLOS One Editor

Re: Response to reviewers

Dear Editor,

Thank you for allowing a resubmission of our manuscript, with an opportunity to address the reviewers’ comments.

We are uploading (a) our point-by-point response to the comments (below) (response to reviewers), (b) an updated manuscript with yellow highlighting indicating changes, and (c) a clean updated manuscript without highlights (PDF main document) along with Latex Source files.

Best regards,

Dr. Atiq Ur Rehman

**Editor Comments**

5. We note that Figure 4 and 8 in your submission contain copyrighted images.

6. We note that Figure 1, 2, 3, 8, 9, 10 and 13 in your submission contain [map/satellite] images which may be copyrighted.

**Author Response**

We are thankful to the reviewer for bringing attention to copyright issues in some figures of the manuscript. The images used in these figures were originally sourced from Google Earth. Since Google earth does not provide its contents to be published under CC 4.0, we have replaced them with alternative options.

**Author Action**

**Figure 1**

Figure 1 has been rectified and Google Earth Images are replaced with administrative boundaries.

**Figure 2:**

Copyright images and logos from Figures 2 are replaced with text and satellite images are removed. The remaining images encapsulated inside the figure belong to our developed GeoSurvey mobile Application and Web-based portal (https://geosurveyapp.com).

**Figure 3:**

This figure also comprises of screenshot of our developed mobile and web-based application for surveys (https://geosurveyapp.com). The satellite images from the figure are replaced.

**Figure 4:**

Figure 4 has been rectified and the satellite images are replaced with markings.

**Figure 8:**

The figure has been rectified and Google Earth logo has been removed.

**Figure 9:**

The Images in figure 9 belong to our experimental setup for Image registration. The satellite images correspond to Sentinel-2 and Planet-Scope and were pivotal for presenting the results. However, since no alternative option can be used for replacing the satellite images used it has been removed from the manuscript Therefore, we request the Editor to allow them in our Manuscript.

**Figure 10:**

Figure 10 has been replaced with an alternative figure, and Google Earth Images has been removed.

**Figure 13:**

Figure 13 has been rectified by removing Google Earth Image.

Moreover, the funding information from the Manuscript has been removed.

**Reviewers Comments**

**Reviewer 1:**

Reviewer #1: This research deals with the topic of Agricultural Remote Sensing in terms of overcoming obstacles in enhancing smallholder economies' agricultural monitoring to mitigate losses such as diminishing farm sizes, lack of reliable data sets, and high costs related to commercial satellite imagery using novel approaches.

Besides the previously recognized challenges for smallholder agriculture, including lack of reliable datasets, geometric error in multi-sensor satellite images, land fragmentation resulting in holdings diminishing in size, inadequate spatial resolution of open data satellites for the field sizes, clouds that can hinder the capturing of satellite images authors emphasize problems specific for country of Pakistan. Namely, the agricultural production of this country contributes 20-22% of its gross domestic product (GDP) to its economy. With a growing population of over 230 million, it is becoming difficult to sustain its own agricultural production, allowing imports of various agricultural commodities. Also, another problem caused by over-hoarding by stakeholders is the growth of counterfeit crops and unlicensed or unaccounted growth of cash crops like tobacco. The country's lack of state-of-the-art technological machinery and the persistence of outdated farming methods are major causes of low yields and financial struggles for smallholders, together making a pivotal point of the described experimentation.

The possible solution for addressing challenges associated with remote sensing-based crop mapping in smallholder agriculture farms includes open source tools and data used for inter and intra-sensor image registration, Segment Anything Model for Geospatial (SAM-GEOs) for delineated vegetation parceling, and a Bidirectional-Long Short-Term Memory-based (Bi-LSTM) deep learning model, developed and trained for crop classification. The proposed method achieves respectable results with accuracy of more than 94% and 96% for validation sets of two data sets collected in the field from the area covering 4 major districts of Pakistan, including a total area of 580000 hectares, during 2 growing seasons.

The manuscript describes a technically sound piece of scientific research with data that supports the conclusions. The experiments have been conducted rigorously, with appropriate controls, replication, and sample sizes. The manuscript presented in an intelligible fashion and written in standard English, but with some typographical errors, that should be corrected. However, manuscripts have some minor shortcomings that should be removed. Although the manuscript contains names of the satellites from which images are used, the direct link to these images is missing. Also, authors should make link to a numerical data .csv file derived from satellite images. The author's motivation for choosing the Bi-LSTM method is not stated in the paper, i.e. ignoring other methods previously applied in this area. Also, the precision measures obtained in this research are slightly worse than in earlier research that used the same method. The existing research could be expanded by determining the reasons for the stated deviation, because 2-3% precision could be high considering the share of agriculture in the total GDP.

1. **Reviewer 1 Comment # 1**

There are typographical errors in the Manuscript.

**Author Response**

Thanks to the reviewer for identifying typographical errors in the manuscript. The grammatical errors have been corrected.

2. **Reviewer 1 Comment # 2**

Although the manuscript contains names of the satellites from which images are used, the direct link to these images is missing.

**Author Response**

The data used for this research is based on the European Space Agency’s Sentinel-2 and Planet Inc. Planet-Scope. The data of Sentinel-2 is open for research and can be retrieved from https://dataspace.copernicus.eu/browser/.Planet-Scope is proprietary and is a fleet of commercial satellite imagery which can be retrieved from <https://www.planet.com>. As recommended the by the reviewer the manuscript is updated with the Tile Ids of Sentinel-2 and Planet-Scope, used in this research

Here are the list of Sentinel-2 Ids used in this research

**Author Action**

The Manuscript is updated via a table for these data IDs.

3. **Reviewer 1 Comment # 3**

Also, authors should make link to a numerical data .csv file derived from satellite images

**Author Response**

The data has been shared through the public repository of Github.

<https://github.com/khanwaleed1011/Training-Data-CSVs-for-PLOSOne/tree/main>

4. **Reviewer 1 Comment # 4**

The author's motivation for choosing the Bi-LSTM method is not stated in the paper, i.e. ignoring other methods previously applied in this area.

**Author Response**

We thank the reviewer for pointing out the missing section in the manuscript.

LSTM is one of the most used techniques in Recurrent Neural Networks (RNNs) for sequential or time series data. Therefore, LSTM allows the evaluation of plantations’ phenological variation, detecting pixel coherence between time sequence data. Among RNNs, the LSTM model is widely used to capture time correlation efficiently. Bidirectional-LSTM is a modified version of LSTM, which learns from the data from both directions instead of one, thus making it more susceptible to crop growth parameters. Bi-LSTM models are more effective as their output depends on the previous and the next segment, in contrast to the unidirectional LSTM models. Many studies have been carried out to improve the LSTM architecture. One of the most powerful approaches is Bi-LSTM. Bi-LSTM models are generally more effective when handling contextual information since their output at a given time depends on both previous and next. Bi-LSTM is selected for its outstanding ability to effectively capture and model temporal dependencies within sequential data from both sides, aligning seamlessly with the dynamic characteristics of agricultural landscapes. While some of the other popular supervised machine learning approaches include Random Forest (RF); Support Vector Machines (SVM) and K-Nearest Neighbor (KNN). RF learns data through binary trees, with low number of parameters, but with a huge variation in results. SVM is by nature a binary classified but is also used for multi class classification. The approach categorizes using linear or nonlinear functions and has a number of parameters to be tuned. KNN is a classic and widely used algorithm for remote sensing. This approach does not include the training phase and is based on majority vote mechanism for the provided data, using euclidean distance.

The decision to employ the Bidirectional-Long Short-Term Memory (Bi-LSTM) method in this study is based on a deliberate assessment of the unique features present in agricultural remote sensing data.

**Author Action**

The manuscript is updated with the following changes highlighting the motivation behind using Bi-LSTM while briefly highlighting the traditional machine learning approaches. Furthermore, more related work on the use of BI-LSTM for Remote Sensing Applications is added to the work.

*Introduction*

***Related Work***

[1]	H. Ijabs and Ē. Urtāns, “Bidirectional Long Short-Term Memory Networks for Automatic Crop Classification at Regional Scale using Tabular Remote Sensing Time Series.,” *Baltic Journal of Modern Computing*, vol. 10, no. 4, 2022.

[2]	H. Crisóstomo de Castro Filho *et al.*, “Rice crop detection using LSTM, Bi-LSTM, and machine learning models from Sentinel-1 time series,” *Remote Sensing*, vol. 12, no. 16, p. 2655, 2020.

[3]	M. Pal, “Random forest classifier for remote sensing classification,” *International journal of remote sensing*, vol. 26, no. 1, pp. 217–222, 2005.

[4]	Z. H. Kok, A. R. M. Shariff, M. S. M. Alfatni, and S. Khairunniza-Bejo, “Support vector machine in precision agriculture: a review,” *Computers and Electronics in Agriculture*, vol. 191, p. 106546, 2021.

[5]	H. Kerner, C. Nakalembe, and I. Becker-Reshef, “Field-level crop type classification with k nearest neighbors: A baseline for a new Kenya smallholder dataset,” *arXiv preprint arXiv:2004.03023*, 2020.

[6]	E. Portalés-Julià, M. Campos-Taberner, F. J. García-Haro, and M. A. Gilabert, “Assessing the sentinel-2 capabilities to identify abandoned crops using deep learning,” *Agronomy*, vol. 11, no. 4, p. 654, 2021.

5. **Reviewer Comment 1 # 5**

The precision measures obtained in this research are slightly worse than in earlier research that used the same method. The existing research could be expanded by determining the reasons for the stated deviation, because 2-3% precision could be high considering the share of agriculture in the total GDP.

**Author Response**

There are several reasons for variation in actuaries compared to the existing research.

1. The dataset used in our study was collected through a rigorous process of ground truth surveys using our developed GeoSurvey application. The survey data employed in this research is split on a field-by-field basis, ensuring the non-convergence of training and testing pixels. This approach differs from traditional methods of splitting the training and testing data for classification, which typically involves pixel-based isolation. In the traditional method, pixels in a vegetation farm are split into training and testing sets. During the testing phase, pixels from a single vegetation farm are used for both training and testing, which can automatically lead to better validation accuracy. In our case, we completely isolated the collected data, ensuring that each training dataset has separate vegetation farms, and each testing dataset has separate vegetation farms. Thus the accuracy achieved highlights the high performance of the classifier on unseen data.
2. The research is performed on a large geographical scale and a large dataset is collected over time having diverse vegetation, focused on smallholder agriculture, which also contributes to slightly lower accuracy than other research performed on relatively larger field sizes where slighted spatial mixing is not an issue.

A brief overview of this method is already provided in “Methods” section of the manuscript.

**Reviewer 2:**

Authors wrote interesting papers about Crop Classification in Smallholder Agriculture using deep learning techniques. Comments and questions which can be discussed in following versions are:

- Introduction is quite big, but interesting. Some parts can be moved to “related work” section
- You used Bi-LSTM DL model. Have you tried to run same model with same dataset using classic LSTM model? Can you compare results (precision and similar) and execution time?
- You mentioned different satellites. Can you compare their resolution/ratio on one place (especially newer versions (like Sentiel-1/2 …) and their dataset size?
- Model parameters are shown at “Model specification” section. Have you tried model with different parameters? Can you compare results (precision and similar) and execution time?

Development of mobile and web-based application for utilizing for collecting and curating these data and the presentation of the results is very praiseworthy.

1. **Reviewer 2 Comment # 1**

Introduction is quite big, but interesting. Some parts can be moved to “related work” section

**Author Response**

Thank you for your comments and suggestions. As recommended, we have moved a section of the introduction to Related Work.

**Author Action**

The following section has been moved from introduction to related Works.

2. **Reviewer 2 Comment # 2**

You used Bi-LSTM DL model. Have you tried to run the same model with the same dataset using the classic LSTM model? Can you compare results (precision and similar) and execution time?

**Author Response**

Thank you for your valuable comment. We have tried the same model with classic LSTM for both *Dataset A* and *Dataset B*. Since both the models are almost same with the BI-LSTM having analyzing data from both sides instead of one, using LSTM a validation accuracy of **94.36%** was achieved for *Dataset A* and **93.75%** for *Dataset B* , which shows a slightly deficient accuracy compared to BILSTM results of **96.82%** for *Dataset A* and **94.62%** for *Dataset B*. A table is provided for the reviewer’s reference.

Table 1 Comparison of LSTM vs Bi-LSTM

|  | **Bi-LSTM** | | **LSTM** | | **% Difference in Validation** |
| --- | --- | --- | --- | --- | --- |
|  | Training Accuracy | Validation Accuracy | Training Accuracy | Validation Accuracy |  |
| Dataset A | 97.61% | 96.82% | 93.66% | 94.36% | -2.46% |
| Dataset B | 94.76 | 94.62 | 93.66% | 93.75% | -0.87% |

**Dataset A**:

In contrast to the BiLSTM, the LSTM model displays a slightly divergent training pattern. Commencing with a training accuracy of 66.2%, the LSTM model incrementally enhances its performance across epochs, ultimately reaching a pinnacle of 95.1%. Although the LSTM model attains a high accuracy level, it seems to converge at a comparatively slower rate in comparison to the BiLSTM. The validation accuracy also demonstrates improvement, initiating at 75.4% and peaking at 94.4%, indicating effective generalization. Both the training and validation loss curves for the LSTM model exhibit a consistent decline, showcasing the model's adeptness at error minimization. The comparison of the BiLSTM and LSTM implies that the former captures more intricate patterns, leading to a swifter and potentially more resilient convergence. Nevertheless, the LSTM model still exhibits robust performance, particularly when considering the intricacies of the task at hand.

|  | Training Accuracy | Validation Accuracy |
| --- | --- | --- |
| 1 | 0.662396 | 0.754224 |
| 2 | 0.796776 | 0.805226 |
| 3 | 0.826689 | 0.81165 |
| 4 | 0.83936 | 0.811984 |
| 5 | 0.845451 | 0.815285 |
| 6 | 0.849241 | 0.820648 |
| 7 | 0.85204 | 0.819627 |
| 8 | 0.855396 | 0.824047 |
| 9 | 0.859063 | 0.829607 |
| 10 | 0.862332 | 0.833969 |
| 11 | 0.866277 | 0.832534 |
| 12 | 0.869578 | 0.836405 |
| 13 | 0.872345 | 0.8422 |
| 14 | 0.876167 | 0.845835 |
| 15 | 0.87851 | 0.852574 |
| 16 | 0.88051 | 0.855717 |
| 17 | 0.882678 | 0.857132 |
| 18 | 0.884939 | 0.859096 |
| 19 | 0.889281 | 0.882633 |
| 20 | 0.893021 | 0.888173 |
| 21 | 0.897719 | 0.886837 |
| 22 | 0.901107 | 0.898527 |
| 23 | 0.903838 | 0.898193 |
| 24 | 0.905112 | 0.900098 |
| 25 | 0.907628 | 0.902083 |
| 26 | 0.909326 | 0.903163 |
| 27 | 0.911075 | 0.904126 |
| 28 | 0.912892 | 0.906267 |
| 29 | 0.915084 | 0.911297 |
| 30 | 0.916659 | 0.913124 |
| 31 | 0.917801 | 0.914067 |
| 32 | 0.920413 | 0.919804 |
| 33 | 0.922221 | 0.918527 |
| 34 | 0.923983 | 0.923654 |
| 35 | 0.926691 | 0.92389 |
| 36 | 0.928623 | 0.926542 |
| 37 | 0.932047 | 0.930452 |
| 38 | 0.933148 | 0.93387 |
| 39 | 0.935161 | 0.936483 |
| 40 | 0.937677 | 0.938684 |
| 41 | 0.939622 | 0.941041 |
| 42 | 0.941915 | 0.941159 |
| 43 | 0.943294 | 0.943084 |
| 44 | 0.944709 | 0.944971 |
| 45 | 0.94596 | 0.945815 |
| 46 | 0.947015 | 0.947348 |
| 47 | 0.947987 | 0.947917 |
| 48 | 0.949572 | 0.949745 |
| 49 | 0.949741 | 0.946955 |
| 50 | 0.950526 | 0.943615 |

|  | Training Loss | Validation Loss |
| --- | --- | --- |
| 1 | 0.988832 | 0.808637 |
| 2 | 0.566614 | 0.656365 |
| 3 | 0.499145 | 0.627577 |
| 4 | 0.466971 | 0.60854 |
| 5 | 0.44813 | 0.605343 |
| 6 | 0.434924 | 0.59212 |
| 7 | 0.423829 | 0.574662 |
| 8 | 0.413252 | 0.564606 |
| 9 | 0.403151 | 0.545955 |
| 10 | 0.394297 | 0.519364 |
| 11 | 0.384786 | 0.503223 |
| 12 | 0.37358 | 0.484465 |
| 13 | 0.364304 | 0.467447 |
| 14 | 0.355363 | 0.45583 |
| 15 | 0.343451 | 0.436159 |
| 16 | 0.335091 | 0.420614 |
| 17 | 0.324749 | 0.399587 |
| 18 | 0.312023 | 0.380603 |
| 19 | 0.298249 | 0.363119 |
| 20 | 0.288201 | 0.354347 |
| 21 | 0.277258 | 0.355264 |
| 22 | 0.270154 | 0.327067 |
| 23 | 0.263377 | 0.303817 |
| 24 | 0.257001 | 0.3076 |
| 25 | 0.25202 | 0.291821 |
| 26 | 0.24757 | 0.290946 |
| 27 | 0.242259 | 0.286177 |
| 28 | 0.237996 | 0.2736 |
| 29 | 0.233749 | 0.274386 |
| 30 | 0.231107 | 0.27 |
| 31 | 0.226777 | 0.270365 |
| 32 | 0.222712 | 0.255156 |
| 33 | 0.218038 | 0.251205 |
| 34 | 0.215372 | 0.245787 |
| 35 | 0.210305 | 0.235728 |
| 36 | 0.206175 | 0.233198 |
| 37 | 0.199557 | 0.218144 |
| 38 | 0.196881 | 0.215523 |
| 39 | 0.191977 | 0.208624 |
| 40 | 0.18655 | 0.198058 |
| 41 | 0.182006 | 0.196928 |
| 42 | 0.177842 | 0.186305 |
| 43 | 0.174668 | 0.190745 |
| 44 | 0.171251 | 0.184576 |
| 45 | 0.167618 | 0.17315 |
| 46 | 0.16516 | 0.170689 |
| 47 | 0.161871 | 0.168419 |
| 48 | 0.159184 | 0.166726 |
| 49 | 0.157668 | 0.173112 |
| 50 | 0.155206 | 0.188606 |

The LSTM model for *Dataset A* also demonstrates good performance, achieving an overall accuracy of 94.36%. It showcases precision, recall, and F1-score metrics for specific classes like 'Urban,' 'Sugarcane,' and 'Water,' attaining precision scores of 98%, 97%, and 100%, respectively. Nevertheless, challenges arise in accurately classifying 'Reed' and 'Rice,' evident in lower precision, recall, and F1-score values compared to BiLSTM. While both models proficiently categorize land cover types, the BiLSTM model appears to hold a slight advantage, particularly in terms of precision and overall average metrics. The classification of model trained with LSTM on *Dataset A* is provided in below.

| **Class** | **Urban** | **Other  Vegetation** | **Maize** | **Reed** | **Rice** | **Sugercane** | **Trees** | **Water** | **Macro  average** | **Weighted  average** |  |
| --- | --- | --- | --- | --- | --- | --- | --- | --- | --- | --- | --- |
| **Precision** | 0.98 | 0.93 | 1 | 0.63 | 0.62 | 0.97 | 0.89 | 1 | 0.88 | 0.95 | **Validation  Accuracy** |
| **Recall** | 1 | 0.92 | 0.85 | 0.57 | 0.87 | 0.95 | 0.93 | 1 | 0.89 | 0.94 | **0.9436** |
| **F1-score** | 0.99 | 0.93 | 0.92 | 0.6 | 0.73 | 0.96 | 0.91 | 1 | 0.88 | 0.94 | **Validation Loss** |
| **Support** | 7282 | 5516 | 3262 | 1917 | 1915 | 13329 | 2788 | 14891 | 50900 | 50900 | **0.1621** |

**Dataset B**

Unlike the Bi-LSTM model, the LSTM model has a different way of performing. Although it gets better during training, reaching a highest accuracy of 93.66%, it's still lower than what the Bi-LSTM model achieved. The validation accuracy goes up to 93.75%, following a similar pattern. While the LSTM model is okay in terms of accuracy, the difference between training and validation accuracy suggests it might be getting too good at the training data (overfitting) but looking at the loss numbers, the training loss keeps dropping, but the validation loss stops at 0.1838. This hints that the LSTM model might struggle to apply what it learned to new data as well as the Bi-LSTM model. So, in short, the LSTM model does well but doesn't quite match the accuracy and adaptability of the Bi-LSTM model for *Dataset B* as well.

|  | Training Accuracy | Validation Accuracy |
| --- | --- | --- |
| 1 | 0.619989 | 0.674427 |
| 2 | 0.730162 | 0.821726 |
| 3 | 0.807971 | 0.837579 |
| 4 | 0.829564 | 0.852976 |
| 5 | 0.852587 | 0.87466 |
| 6 | 0.874231 | 0.889696 |
| 7 | 0.88753 | 0.904675 |
| 8 | 0.898102 | 0.913556 |
| 9 | 0.90831 | 0.924394 |
| 10 | 0.914465 | 0.93203 |
| 11 | 0.918915 | 0.933331 |
| 12 | 0.921735 | 0.935165 |
| 13 | 0.923354 | 0.941111 |
| 14 | 0.925814 | 0.9398 |
| 15 | 0.92663 | 0.942915 |
| 16 | 0.927702 | 0.942108 |
| 17 | 0.927936 | 0.941946 |
| 18 | 0.929262 | 0.942184 |
| 19 | 0.92925 | 0.940997 |
| 20 | 0.930123 | 0.944046 |
| 21 | 0.930779 | 0.94263 |
| 22 | 0.931164 | 0.942953 |
| 23 | 0.93157 | 0.945385 |
| 24 | 0.931693 | 0.943257 |
| 25 | 0.932352 | 0.943362 |
| 26 | 0.932774 | 0.942896 |
| 27 | 0.933233 | 0.943542 |
| 28 | 0.933025 | 0.944273 |
| 29 | 0.933469 | 0.944273 |
| 30 | 0.933554 | 0.942925 |
| 31 | 0.934162 | 0.94018 |
| 32 | 0.933984 | 0.941899 |
| 33 | 0.934305 | 0.9451 |
| 34 | 0.934415 | 0.944008 |
| 35 | 0.934286 | 0.939325 |
| 36 | 0.934961 | 0.945698 |
| 37 | 0.9354 | 0.944302 |
| 38 | 0.93502 | 0.943257 |
| 39 | 0.935431 | 0.944368 |
| 40 | 0.935423 | 0.945014 |
| 41 | 0.935144 | 0.939923 |
| 42 | 0.935783 | 0.945698 |
| 43 | 0.935963 | 0.94529 |
| 44 | 0.936146 | 0.943466 |
| 45 | 0.936138 | 0.94491 |
| 46 | 0.936062 | 0.939477 |
| 47 | 0.936332 | 0.938812 |
| 48 | 0.936509 | 0.945489 |
| 49 | 0.936551 | 0.945413 |
| 50 | 0.936672 | 0.937587 |

|  | Training Loss | Validation Loss |
| --- | --- | --- |
| 1 | 0.909098 | 0.726362 |
| 2 | 0.603661 | 0.480633 |
| 3 | 0.483903 | 0.43684 |
| 4 | 0.439283 | 0.394448 |
| 5 | 0.387237 | 0.332242 |
| 6 | 0.33963 | 0.29574 |
| 7 | 0.311106 | 0.263812 |
| 8 | 0.288476 | 0.245044 |
| 9 | 0.267753 | 0.215945 |
| 10 | 0.252262 | 0.197301 |
| 11 | 0.241163 | 0.191151 |
| 12 | 0.233072 | 0.2058 |
| 13 | 0.228271 | 0.180311 |
| 14 | 0.222008 | 0.175068 |
| 15 | 0.219804 | 0.174056 |
| 16 | 0.216484 | 0.178339 |
| 17 | 0.214803 | 0.180315 |
| 18 | 0.21163 | 0.179135 |
| 19 | 0.209917 | 0.180935 |
| 20 | 0.207381 | 0.168874 |
| 21 | 0.205577 | 0.168259 |
| 22 | 0.205133 | 0.165352 |
| 23 | 0.202892 | 0.166385 |
| 24 | 0.201807 | 0.17203 |
| 25 | 0.200221 | 0.167405 |
| 26 | 0.199946 | 0.174793 |
| 27 | 0.197683 | 0.172533 |
| 28 | 0.19876 | 0.168185 |
| 29 | 0.195933 | 0.167731 |
| 30 | 0.195339 | 0.172763 |
| 31 | 0.195557 | 0.187278 |
| 32 | 0.193901 | 0.178806 |
| 33 | 0.193137 | 0.165866 |
| 34 | 0.19254 | 0.168971 |
| 35 | 0.192527 | 0.184789 |
| 36 | 0.191269 | 0.165058 |
| 37 | 0.190043 | 0.17078 |
| 38 | 0.190656 | 0.173727 |
| 39 | 0.18943 | 0.170062 |
| 40 | 0.189138 | 0.169325 |
| 41 | 0.188919 | 0.190082 |
| 42 | 0.187478 | 0.165623 |
| 43 | 0.187093 | 0.169317 |
| 44 | 0.186434 | 0.174976 |
| 45 | 0.18652 | 0.172261 |
| 46 | 0.186543 | 0.19135 |
| 47 | 0.186241 | 0.191551 |
| 48 | 0.18543 | 0.166611 |
| 49 | 0.185219 | 0.168939 |
| 50 | 0.183826 | 0.196081 |

The LSTM model for *Dataset B* demonstrates acceptable performance in the classification report, achieving good precision, recall, and F1-score values across Tobacco, Other Vegetation, Urban, and Water categories. Although the precision and recall for the Urban class are somewhat lower compared to Bi-LSTM, the overall performance remains satisfactory. Notably, the Water class achieves perfect precision and a respectable recall, indicating the model's competence in identifying water-related features. The LSTM model achieves an overall accuracy of 93.75%, precision of 93.99%, recall of 93.75%, and F1-score of 93.71%. While these metrics slightly trail behind those of the BiLSTM model, they exhibit the LSTM model's capability in classifying different features within the dataset.

| **Class** | **Tobacco** | **Other  Vegetation** | **Urban** | **Water** | **Macro  average** | **Weighted  average** |  |
| --- | --- | --- | --- | --- | --- | --- | --- |
| **Precision** | 0.93 | 0.93 | 0.96 | 1 | 0.96 | 0.94 | **Validation  Accuracy** |
| **Recall** | 0.92 | 0.97 | 0.87 | 0.97 | 0.93 | 0.94 | **0.9375** |
| **F1-score** | 0.92 | 0.95 | 0.92 | 0.99 | 0.94 | 0.94 | **Validation Loss** |
| **Support** | 22835 | 59057 | 19425 | 3965 | 105282 | 105282 | **0.196** |

**Author Action**

Details of these LSTM and Bi-LSTM is added to the Manuscript.

3. **Reviewer 2 Comment # 3**

You mentioned different satellites. Can you compare their resolution/ratio on one place (especially newer versions (like Sentinel-1/2 …) and their dataset size?

**Author Action**

A table has been added to the manuscript providing the frequently used remote sensing satellites in vegetation monitoring.

4. **Reviewer 2 Comment # 4**

Model parameters are shown at “Model specification” section. Have you tried model with different parameters? Can you compare results (precision and similar) and execution time?

**Author Response**

We thank the reviewer for his insight into results and appreciate his/her query. For the model training results are provided in the table, for different hyperparameter tuning.

The presented results in the manuscript are the result of rigorous training and testing of different parameters on BI-LSTM, and hence presents the best results achieved in terms of Training accuracy, Validation accuracy, Training Loss, Validation Loss, and their respective trends during model training.

1. Training and Testing Curves during model training
2. Eliminating overfitting in the model.

As suggested by the reviewer we present a comparison of different hyperparameter from the literature and present our finding in the form of Table and Figure. The results identify best hyperparameters for Dataset A and Dataset B while taking into consideration.

a. Training and Validation Accuracy
b. Training and Validation Loss

The following experiments by tuning different hyperparameters have been conducted. These filters include Activation Function, Filters, Optimizer and Number of Layers as shown in the table below. The best combination for both *Dataset A* and *Dataset B* using BI-LSTM after testing are highlighted in green.

The execution time required for model training for different sets of hyperparameters varies with different runs with different machines. Our experiments were performed on Precision 7820 Tower, powered by Intel Xeon Silver 4208, 64 Gigabyte of RAM and Nvidia Quadro RTX 8000 with a 48GB of memory. Also weight initialization is kept random which also contributes to changing total model training time.

| **Activation Function** | sigmoid | softplus | softsign | tanh | selu | elu | relu |
| --- | --- | --- | --- | --- | --- | --- | --- |
| **Filters** | 8 | 16 | 32 | 64 | 128 |  |  |
| **Optimizer** | SGD | Nadam | Adagrade | Adadelta | Adamax | Adam |  |
| **Layers** | 1 | 2 | 3 | 4 |  |  |  |

Table 2. Grid Search for Optimum Hyperparameter Analysis

|  |  | ***Dataset A*** | | | ***Dataset B*** | | |
| --- | --- | --- | --- | --- | --- | --- | --- |
|  |  | Training | Validation | Execution Time in secs | Training | Validation | Execuation Time |
| **Activation Function** (Layers: 2, Filters: 64, Optimizer: Adam) | Sigmoid | 63.08 | 55.44 | 407.56 | 63.76 | 72.12 | 616.29 |
|  | Softplus | 91.32 | 88.43 | 386.3 | 93.62 | 94.46 | 630.5 |
|  | Softsign | 93.74 | 90.7 | 376.43 | 93.9 | 94.31 | 754.13 |
|  | Tanh | 94.8 | 92.9 | 369.57 | 94.08 | 94.53 | 593.75 |
|  | Selu | 96.59 | 94.93 | 396.34 | 94.08 | 94.65 | 662.39 |
|  | elu | 94.7 | 92.39 | 373.6 | 93.93 | 94.54 | 664.09 |
|  | Relu | 97.61 | 96.82 | 471.5 | 94.76 | 94.62 | 670.954 |
| **Filters**  (Activation Function Relu, 2 Layers, 64 filters | 8 | 85.77 | 88.74 | 440.56 | 83.2 | 89.25 | 573.52 |
|  | 16 | 93.15 | 92.14 | 610.13 | 92.05 | 94.4 | 621.46 |
|  | 32 | 97.36 | 96.51 | 468.917 | 93.47 | 94.64 | 649.25 |
|  | 64 | 97.61 | 96.82 | 471.5 | 94.76 | 94.62 | 670.954 |
|  | 128 | 95.23 | 92.8 | 476.5 | 94.39 | 94.69 | 697.2 |
| **Optimization  Function** (Activation  Function: Relu, Layer: 2 Adam, Filters: 64 | SGD | 60.94 | 55.44 | 519.66 | 59.28 | 56.09 | 752.6 |
|  | Nadam | 94.21 | 91.41 | 485.722 | 94.15 | 94.31 | 1,421 |
|  | Adagrad | 66.09 | 55.44 | 480.39 | 59.17 | 56.09 | 1095.87 |
|  | Adadelta | 55.61 | 54.26 | 491.63 | 59.16 | 56.09 | 789 |
|  | Adamax | 91.28 | 89.45 | 497.187 | 93.02 | 94.63 | 851.55 |
|  | Adam | 97.61 | 96.82 | 471.5 | 94.76 | 94.62 | 670.954 |
| **Layers** (Activation  Function: Relu, Optimization  function: Adam, Filters: 64) | 1 | 93.93 | 92.14 | 361.23 | 94.22 | 94.89 | 497.59 |
|  | 2 | 97.61 | 96.82 | 471.5 | 94.76 | 94.62 | 670.954 |
|  | 3 | 96.83 | 94.4 | 548.56 | 94.22 | 94.48 | 1163.67 |
|  | 4 | 94.2 | 92.29 | 851.79 | 94.15 | 94.45 | 1,360.50 |

In this comprehensive evaluation of model configurations on Datasets A and B, the investigation primarily centers around identifying optimal hyperparameter combinations based on Training accuracy, Validation Accuracy, with due consideration to Training Loss and Validation Loss. After detailed analysis, it is noted that the activation function "Relu" consistently yields better performance across various scenarios. Notably, when employing 2 layers with 64 filters and the Adam optimizer, the Relu activation function attains the highest Training and Validation Accuracy, reaching 97.61% and 96.82%, respectively. For Dataset A, the activation function "Relu" consistently yields the lowest Training and Validation Loss when applied with 2 layers, 64 filters, and the Adam optimizer, recording values of 0.0783 and 0.1038, respectively. Moreover, the performance across different numbers of filters and layers further reinforces the superiority of the Relu activation function. Thus, based on the outcomes presented in the table, it can be inferred that the Relu activation function, in conjunction with 2 layers, 64 filters, and the Adam optimizer, constitutes the optimal hyperparameter combination, demonstrating superior accuracy and efficiency in both training and validation phases.

Upon scrutinizing the dataset B results, a parallel trend emerges in favor of the Relu activation function. In concordance with Dataset A, the 2 layers, 64 filters, and Adam optimizer configuration, coupled with Relu activation, continues to exhibit best performance. Specifically, the highest Training and Validation Accuracy is achieved with Relu, recording values of 94.76% and 94.62%, respectively. Similarly, in Dataset B, the Relu activation function, under the same configuration, demonstrates superior performance with the lowest Training and Validation Loss values of 0.1692 and 0.1473, respectively. Furthermore, the exploration of diverse filter quantities and layer variations reinforces the robustness of the Relu activation function within the context of Dataset B. Consequently, mirroring the findings in Dataset A, it can be deduced that the Relu activation function, when applied in tandem with 2 layers, 64 filters, and the Adam optimizer, stands out as the optimal hyperparameter combination for maximizing accuracy while minimizing loss in both training and validation phases on Dataset B.

Furthermore, an exploration of various filter quantities and layer variations within the dataset B context reinforces the consistent performance of the Relu activation function in minimizing Training and Validation Loss. Thus, based on the outcomes presented in the table, it can be inferred that the Relu activation function, when utilized with 2 layers, 64 filters, and the Adam optimizer, emerges as the optimal hyperparameter combination for minimizing both Training and Validation Loss in neural network training scenarios for both Dataset A and Dataset B.

**Author Action**

The manuscript is updated with the following paragraphs and a figure in Model Specification section. Moreover, a comparison table has also been added to the Manuscript.
